# Adaptive integrate-and-fire model reproduces the dynamics of olfactory receptor neuron responses in a moth

**DOI:** 10.1098/rsif.2019.0246

**Published:** 2019-08-07

**Authors:** Marie Levakova, Lubomir Kostal, Christelle Monsempès, Philippe Lucas, Ryota Kobayashi

**Affiliations:** 1 Department of Computational Neuroscience, Institute of Physiology of the Czech Academy of Sciences, Videnska 1083, 14220 Prague 4, Czech Republic; 2 Institute of Ecology and Environmental Sciences, INRA, route de St Cyr, 78000 Versailles, France; 3 Principles of Informatics Research Division, National Institute of Informatics, 2-1-2 Hitotsubashi, Chiyoda-ku, Tokyo, Japan; 4 Department of Informatics, SOKENDAI (The Graduate University for Advanced Studies), 2-1-2 Hitotsubashi, Chiyoda-ku, Tokyo, Japan

**Keywords:** olfactory receptor neuron, integrate-and-fire model, adaptive threshold

## Abstract

In order to understand how olfactory stimuli are encoded and processed in the brain, it is important to build a computational model for olfactory receptor neurons (ORNs). Here, we present a simple and reliable mathematical model of a moth ORN generating spikes. The model incorporates a simplified description of the chemical kinetics leading to olfactory receptor activation and action potential generation. We show that an adaptive spike threshold regulated by prior spike history is an effective mechanism for reproducing the typical phasic–tonic time course of ORN responses. Our model reproduces the response dynamics of individual neurons to a fluctuating stimulus that approximates odorant fluctuations in nature. The parameters of the spike threshold are essential for reproducing the response heterogeneity in ORNs. The model provides a valuable tool for efficient simulations of olfactory circuits.

## 1. Introduction

Many animals rely on olfaction for detecting food, natural predators and mating partners. The odorant is initially recognized by olfactory receptor neurons (ORNs). The information is then transferred to a secondary region, either the antennal lobe in insects or olfactory bulb in vertebrates. Projections from the secondary region extend to higher order brain regions, the mushroom body and lateral horn in insects and the orbitofrontal cortex, amygdala, entorhinal cortex and ventral striatum in vertebrates. The architecture of the olfactory circuit differs from that of other sensory modalities (for a review, see [1,2]); for example, the olfactory circuit consists of fewer layers. Therefore concepts derived from experimental and theoretical studies on other systems may not be applicable to olfaction. Computational models that can replicate the behaviour of real ORNs during odorant stimulation may generate testable hypotheses on mechanisms underlying olfactory transduction and encoding.

Indeed, computational models have enhanced our understanding of the mechanisms underlying odorant detection in both invertebrates and vertebrates [3–8] and facilitated investigations of olfactory pathway functions [9–12]. Such models have also been used to clarify the coding properties of ORNs such as the stimulus–response relationship of the ORNs [13,14] and the implications of the efficient coding hypothesis [15].

Pheromone detection in moth ORNs occurs in two stages: receptor activation by the odorant and action potential (spike) generation. Odorant molecules are first absorbed by the sensillum lymph, where they initiate a cascade of complex biochemical interactions. Receptor activation and related downstream signalling cascades leading to membrane depolarization have been described by various mathematical models [3,14,16], including detailed biophysical models [4–7,17,18]. To understand the mechanisms of pheromone detection, it is essential to develop a computational model that replicates odorant-evoked ORN responses.

Reduced neuronal models, such as the leaky integrate-and-fire (LIF) neuron [19–21], can be good approximations of real neurons [22,23] and therefore useful tools for simulating and investigating prominent features of network dynamics [24,25]. A few models incorporating receptor activation into a simple spike generation mechanism based on the LIF model have been developed [13,26] in order to study steady-state ORN behaviour. However, the LIF model cannot accurately replicate the response dynamics.

Here, we develop a computational model for individual ORNs that generates spikes in response to dynamic odorant stimulation. We demonstrate that an adaptation mechanism in spike threshold is necessary to reproduce the response dynamics of ORNs. The mathematical tractability and simplicity of the proposed model allows for efficient simulations and analysis of ORN spiking activity.

## 2. Results

### 2.1. Typical response of olfactory receptor neurons to pheromone

**Figure 1.**
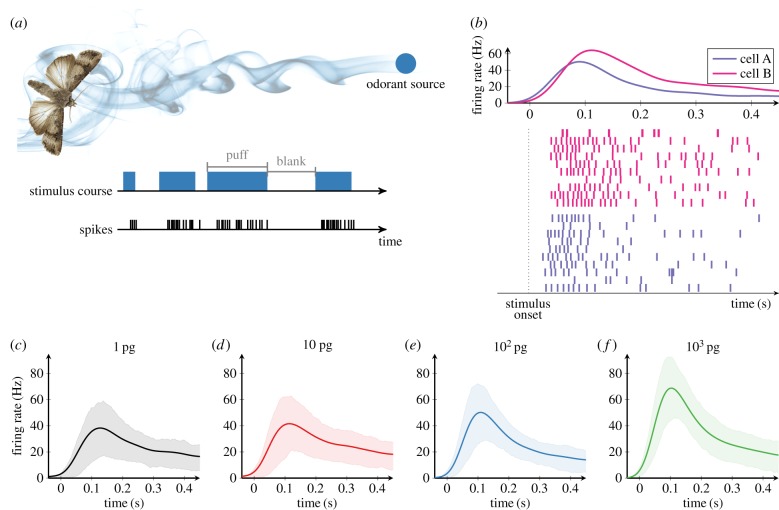
Experimental data for the responses of olfactory receptor neurons (ORNs) to pheromone stimulation. (*a*) ORNs were stimulated by intermittent delivery of the sex pheromone (four pheromone doses ranging from 1 to 1000 pg) to mimic fluctuating odorant concentration in a pheromone plume. (*b*) Examples of spike trains generated by two ORNs (cells A and B) in response to 0.5 s of constant pheromone stimulation at 100 pg. Top: The average firing rate of each cell. Bottom: Raster plots of 10 trials (rows) from each cell. Note the heterogeneity in firing rates between the two ORNs despite stimulation by the same pheromone pulse. (*c*–*f*) The average firing rate across cells in response to the same 0.5 s pulse stimulus of pheromone at different doses (1–1000 pg). The shaded area represents the range between the lower and upper quartile trajectory. (Online version in colour.)

Experimental data were obtained from ORNs by applying different pheromone doses to antennae of the moth *Agrotis ipsilon* (see Methods for details). To simulate the fluctuating odorant concentration in a natural environment [27], the pheromone was applied in short intermittent pulses (*puffs*) separated by stimulus-free periods (*blanks*) of random duration (figure 1*a*).

Responses of different ORNs to the same pheromone pulse exhibited marked cell-to-cell variability (figure 1*b*) as reported in previous studies [28,29]. This response heterogeneity of ORNs might be caused, for example, by differences in the density of olfactory receptors (ORs), odorant-binding proteins and odorant-degrading enzymes among ORNs. Nonetheless, averaged responses across cells demonstrated a typical phasic–tonic time course regardless of pheromone dose (figure 1*c*–*f*). From a baseline rate near 0 Hz, the firing rates increased rapidly (phasic period), reaching a peak around 100 ms after stimulus onset, and then slowly decaying toward a steady-state firing rate that was higher than the spontaneous firing rate (tonic period). The peak firing rate increased with pheromone dose, but the delay of the peak firing rate (latency) and the phasic–tonic response time course did not change.

### 2.2. Model of an olfactory receptor neuron

**Figure 2.**
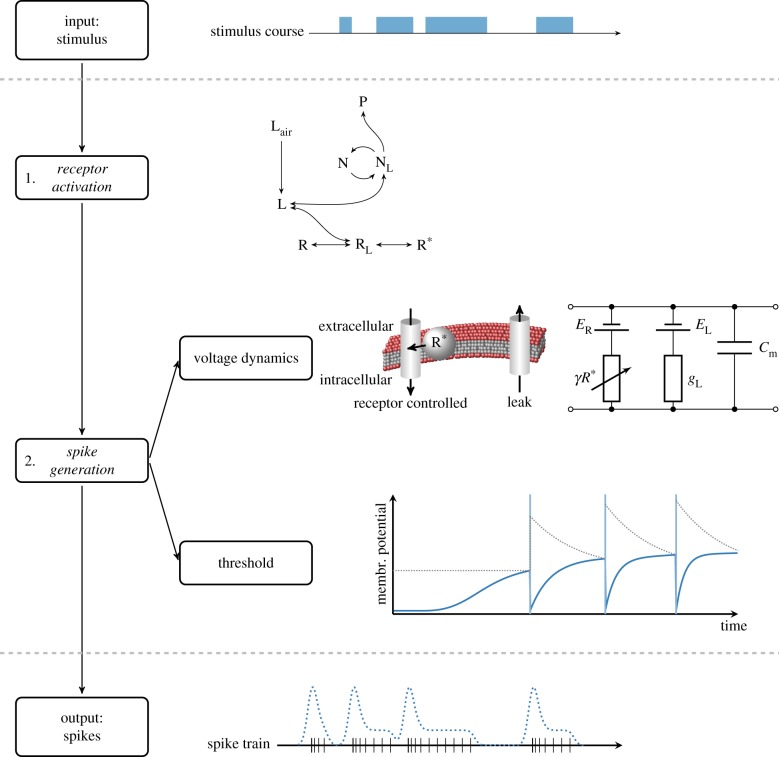
Proposed model of an olfactory receptor neuron (ORN). *Stimulus.* The odorant concentration fluctuating in time is the input to the model neuron. *(1) Receptor activation*. The odorant molecules in the air L_air_ are adsorbed in the lymph at the receptor site. The adsorbed molecules L either bind to receptors R resulting in activated receptors R* or they are degraded by an enzyme N, which converts them into an inactive product P. *(2) Spike generation*. Activated receptors R* induce a receptor current in a single-compartment model. The model neuron generates action potentials when the membrane potential reaches a threshold *θ*(*t*). Note that a time-dependent spike threshold model (dotted) can reproduce experimentally observed ORN responses. *Response.* The model provides spike times from which the firing rate can be calculated. (Online version in colour.)

The proposed ORN model (figure 2) consists of two main parts: (i) *receptor activation* due to pheromone stimulation and (ii) *spike generation* according to an integrate-and-fire mechanism.

*Receptor activation*. We describe the process of receptor activation by the following chemical reactions, derived by Kaissling and coworkers [15,30,31]

2.1 $$L_{\mathrm{air}}\overset{k_{i}}{\to }L$$

2.2 $$nL+R\overset{k_{1}}{\underset{k_{-1}}{⇌}}R_{L}\overset{k_{2}}{\underset{k_{-2}}{⇌}}R^{*}$$

2.3 $$\text{and}\;L+N\overset{k_{3}}{\underset{k_{-3}}{⇌}}N_{L}\overset{k_{4}}{\to }P+N.$$

Equation (2.1) describes an absorption of odorant molecules in the air L_air_ by the sensillum lymph at a rate *k*_*i*_, which yields odorant molecules at the receptor site L. Equation (2.2) describes the binding of *n* molecules of odorant L to a receptor. Odorant molecules L reversibly bind to free receptors R at rates *k*_1_ and *k*_−1_, which yields the receptor–ligand complex R_L_. Then, the complexes R_L_ are reversibly activated (R*) at a rate *k*_2_ and *k*_−2_. Finally, equation (2.3) describes the kinetics of odorant degradation at the receptor site by an odorant degrading enzyme N. The odorant and enzyme reversibly form a complex N_L_ according to rate constants *k*_3_ and *k*_−3_, and the complex is degraded into an inactive product P at a rate *k*_4_. The chemical kinetics (2.1)–(2.3) can be described by a system of differential equations (see Methods, equations (4.1)–(4.6)).

*Spike generation*. We describe the ORN by a single-compartment model. The membrane potential *V*(*t*) evolves according to [32]

2.4 $$C_{m}\frac{\text{d}V}{\text{d}t}=-g_{L}(V-E_{L})+I_{R}(t),$$

where C_*m*_ is the cell capacitance, *g*_*L*_ is the leak conductance and *E_L_* is the reversal potential of the leak current. The current from the odorant receptors *I_R_*(*t*) is determined by the quantity of activated receptors according to [13]

2.5 $$I_{R}(t)=-\gamma R^{*}(t)(V-E_{R}),$$

where R*(*t*) is the concentration of activated receptors R* at time *t*, *E_R_* is the reversal potential of the receptor current and *γ* represents the conductance induced by a single activated receptor R*. A spike is generated when the membrane potential *V*(*t*) reaches a threshold *θ*(*t*). After each spike, the membrane potential is reset to a value *V*_reset_. In the following sections, we consider two types of spike thresholds, a constant threshold and an adaptive threshold.

### 2.3. Model with constant spike threshold cannot reproduce the response dynamics of an olfactory receptor neuron

**Figure 3.**
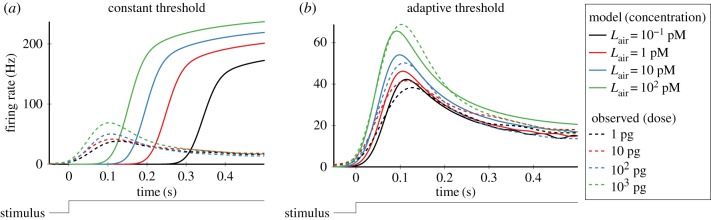
Model with an adaptive spike threshold can reproduce the phasic–tonic response of ORNs to a pulse odorant stimulation. Average responses of ORNs (dashed lines) were compared with the responses of the model neurons (solid lines), i.e. the model with a constant threshold (*a*) and the model with an adaptive threshold (*b*). The unit receptor conductance was *γ* = 41 nS · μM^−1^ in (*a*) and *γ* = 99 nS · μM^−1^ in (*b*). Each spike generated by the model with a constant threshold (*a*) was followed by a 3 ms refractory period. The pheromone concentration in the air, *L*_air_, was set to 0.1 pM, 1 pM, 10 pM, 100 pM for the pheromone doses 1 pg, 10 pg, 100 pg, 1000 pg, respectively. See tables 1 and 2 for the other parameters. (Online version in colour.)

First, we considered the model with a constant spike threshold, *θ*(*t*) = *θ*_0_, known as the leaky integrate-and-fire (LIF) model [32]. We investigated whether the LIF model with receptor dynamics (2.1)–(2.5) can reproduce the average response of ORNs to a pheromone pulse stimulus (figure 1*c*–*f*). We observed that the firing rates of the model increase monotonically, whereas the firing rates of ORNs always exhibited a peak followed by a slower decline to steady state (phasic–tonic response) (figure 3*a*). The model firing rates increase monotonically because the number of activated receptors R*(*t*) increases during the stimulation period. Thus, the model based on (2.1)–(2.5) with a constant spike threshold cannot reproduce the time course of the average ORN response.

**Table 1.** Parameters for the model of receptor activation (equations (2.1)–(2.3)).

	value	unit	fitted/fixed
R_tot_	1.64	μM	fixed [16,30,31]
N_tot_	1	μM	fixed [16,30,31]
*k*_*i*_	10^6^	s^−1^	fixed [37]
*k*_1_	0.209	s^−1^ · μM^−1^	fixed [16,30,31]
*k*_−1_	7.9	s^−1^	fixed [16,30,31]
*k*_2_	16.8	s^−1^	fixed [16,30,31]
*k*_−2_	98	s^−1^	fixed [16,30,31]
*k*_3_	100	s^−1^ · μM^−1^	fixed
*k*_−3_	98.9	s^−1^	fixed [16,30,31]
*k*_4_	40 000	s^−1^	fixed
*n*	0.056		fitted

**Table 2.** Parameters for the model of spike generation.

	value	unit	fitted/fixed
C_*m*_	0.00144	nF	fixed [7,38]
*g*_*L*_	1.44	nS	fixed [7,38]
*γ*	99.27	nS · μM^−1^	fitted
*E_L_*	−62	mV	fixed [7,32,39,40]
*E_R_*	0	mV	fixed [7]
*V*_reset_	−62	mV	fixed [7,32]
*θ*_0_	−55	mV	fixed [32]
Δ	0.77	mV s	fitted
*τ*	0.58	s	fitted

Except for non-decreasing firing rate profiles, the model has another issue of being able to reproduce correctly only either the peak firing rate or the first-spike latency, but not both of them simultaneously. This problem could only be numerically resolved by allowing an unphysiologically long refractory period after each spike. Figure 3*a* shows a compromise fit that could be achieved with a realistic 3 ms refractory period, where both the peak firing rate and the first-spike latency are much larger than in real ORNs.

### 2.4. Model with an adaptive spike threshold reproduces the response dynamics of an olfactory receptor neuron

Since the LIF model with constant spike threshold could not replicate the qualitative characteristics of ORN responses, it was modified by including an adaptive spike threshold [33–36], which depends on previous spike times. The threshold *θ*(*t*) increases by Δ/*τ* after each spike and decreases exponentially to an asymptotic level *θ*_0_ with the time constant *τ*. The parameter Δ represents the strength of adaptation (see Methods for a formal mathematical description).

**Figure 4.**
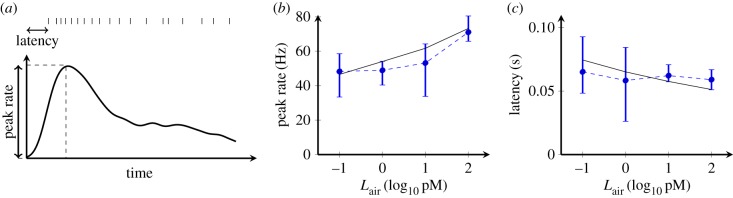
Model with an adaptive spike threshold can reproduce the odorant response characteristics of ORNs. (*a*) A scheme illustrating two salient characteristics of the response time course: the peak firing rate and the first-spike latency. (*b*,*c*) The effect of odorant concentration on the response characteristics. The peak firing rate (*b*) and the first-spike latency (*c*) obtained from experimental data (dashed blue, mean with inter-quartile range) were compared with those obtained from the model (solid black). (Online version in colour.)

Unlike the LIF model, the model with the adaptive spike threshold is able to accurately reproduce the time course of the average ORN responses under each odorant concentration (figure 3*b*). In addition, the model captures the dependence of the response characteristics of ORNs, i.e. the peak firing rate (figure 4*b*) and the first-spike latency (figure 4*c*), on the odorant concentration over a wide range of odorant doses (1000-fold).

The model parameters are summarized in tables 1 and 2. Most of them were adopted from previous studies [7,15,16,30–32,37], while the two rate constants for receptor activation (equation (2.3)), *k*_3_ and *k*_4_, were chosen to achieve rapid deactivation of L. The remaining four parameters (*n*, *τ*, Δ and *γ*) were determined by minimizing the integrated squared error between the average response of ORNs and the model response (see Methods).

### 2.5. Model with an adaptive threshold reproduces responses to a fluctuating stimulus

In the natural environment, odorant concentrations fluctuate rapidly; therefore, it is crucial to replicate the response dynamics of an ORN to such stimulation. To mimic the natural pheromone plume under experimental conditions, we stimulated the antennae by intermittent delivery of the pheromone [41,42]. The firing rates of individual ORNs were then compared with those generated by the model with the adaptive spike threshold.

Since we wanted to reproduce the activity of individual ORNs, we had to take into account a cell-to-cell variability in ORN responses (figure 1*b*). The heterogeneity among ORNs can be captured by fitting some of the model parameters to the experimental recording of each individual ORN (see Methods), while keeping all the other parameters fixed as in tables 1 and 2. As for the choice of which parameters should be allowed to vary across the cells, we tested three options. First, we let *γ* vary (heterogeneity in *γ*); second, we let the pair of threshold parameters Δ and *τ* be cell specific (heterogeneity in (Δ, *τ*)); and third, we fitted all three parameters *γ*, Δ and *τ* to each neuron (heterogeneity in (*γ*, Δ, *τ*)). Finally, we examined the prediction performance of each heterogeneous model by the coefficient of determination (see Methods).

**Figure 5.**
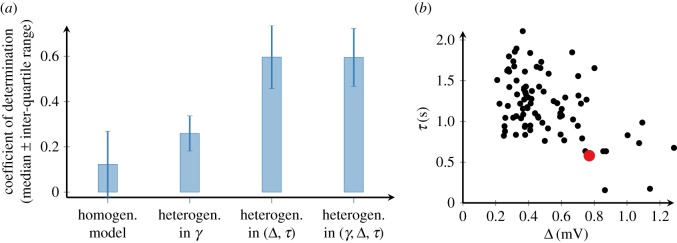
Heterogeneity in ORN model parameters. (*a*) Prediction performance of the model with all parameters fixed (homogeneous model) and three models with heterogeneous parameters (heterogeneity in *γ*, heterogeneity in (Δ, *τ*) and heterogeneity in (*γ*, Δ, *τ*)). (*b*) Scatter plot of the threshold parameters (Δ and *τ*) adjusted to individual neurons. The red dot represents the parameters fitted to the average ORN response (table 2). (Online version in colour.)

The prediction performances of the three heterogeneous models with cell-specific parameters were compared with the model where all parameters were fixed for all cells as in table 2 (homogeneous model); see figure 5*a*. The median prediction performance of the homogeneous model was 0.13 (inter-quartile range: −0.02 to 0.30). Fitting only *γ* led to a mild improvement in the prediction performance (median 0.26, inter-quartile range: 0.18 to 0.35). The prediction performance improved substantially with heterogeneous *τ* and Δ (median 0.6, inter-quartile range: 0.46 to 0.67). Having all three parameters *γ*, *τ*, Δ heterogeneous did not bring any improvement compared with heterogeneity only in (Δ, *τ*) and the median prediction error was even slightly lower (median 0.59, inter-quartile range: 0.47 to 0.66), most likely because too many free parameters led to overfitting.

**Figure 6.**
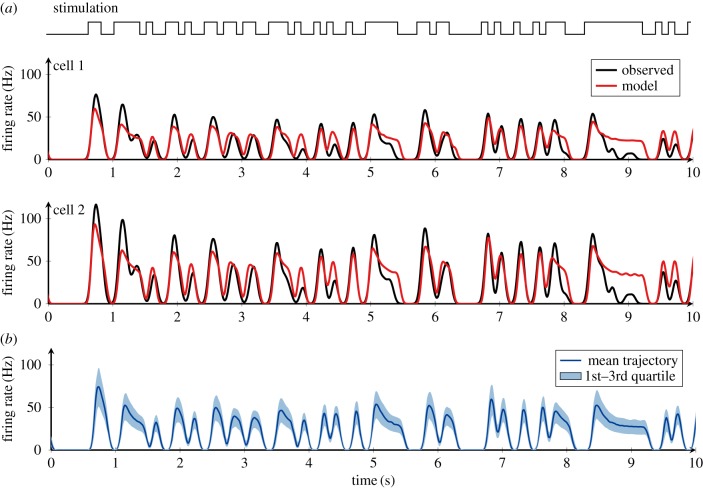
Fit of the model with an adaptive spike threshold to individual ORN responses. (*a*) Top: Time course of the pheromone stimulus. The stimulus was switching between ON and OFF states. In the ON state, the pheromone dose was 100 pg. Bottom: Firing rate time courses of two neurons (cells 1 and 2) obtained from experiments (black) and those of the model with individually tuned threshold parameters (red). (*b*) The distribution of firing rates of the model neurons whose threshold parameters were derived from 84 ORNs. The dark blue line represents the mean trajectory and the light blue area represents the range between the first and the third quartile. The individual trajectories vary only in the amplitude of the fluctuations, not in the temporal pattern. (Online version in colour.)

Therefore, we concluded that the cell-to-cell heterogeneity among ORNs is best captured by fitting the threshold parameters (Δ and *τ*) to the experimental recording of each individual ORN, since this yields a significant improvement in the prediction performance over the homogeneous model (Wilcoxon’s rank sum test, *p* < 0.001, *n* = 84). Figure 6*a* illustrates an example of the model fit to recordings of two neurons. While the temporal pattern of the observed responses is similar, the amplitudes are different. The model with the adaptive spike threshold reproduces the response time course of the two neurons accurately. The distribution of the response time course of the fitted model neurons (*n* = 84) to the same stimulus is shown in figure 6*b*. Owing to the heterogeneity in threshold parameters, the amplitudes of the responses are highly variable among the model neurons, but the temporal patterns of the responses remain similar. Figure 5*a* shows the threshold parameters obtained from all ORNs. The mean values (+/− the standard deviation) of the parameters are 1.2 ± 0.38 s for the threshold time constant *τ* and 0.5 ± 0.23 mV s for the adaptation level Δ. Values of *τ* and Δ are negatively correlated (correlation coefficient −0.48). This finding can be intuitively explained as that these two parameters can compensate for each other to some extent. A similar firing rate may be achieved by combining either a small step increase and a slow relaxation time or a big increase and fast relaxation. Although the threshold parameters exhibit high variability among the ORNs, they are comparable to the parameters fitted to the average response (table 2).

## 3. Discussion

We present a computational model of a moth ORN that reproduces the firing rate dynamics of an ORN under intermittent pheromone stimulation over a 1000-fold range of concentrations. Further, our model captures cell-to-cell response variability of ORNs by tuning only two model parameters controlling the spike threshold. The model is less accurate for longer stimulations, where the model firing rate increases more slowly than the true firing rate. The model also mildly underestimates maximal spike rates.

### 3.1. Heterogeneity of olfactory receptor neurons

The response heterogeneity of moth ORNs, manifested by different dose–response properties among cells, and its impact on neuronal coding were thoroughly studied by Rospars *et al.* [29]. In addition, cell-to-cell response variability among ORNs has been investigated in other animal species such as mice [43]. This variability is captured in our model by setting different threshold parameters, i.e. the strength and the time constant of the adaptation. Previous works [36,44] suggested that the biophysical origins of the adaptive threshold are the slow K^+^ currents in the neuron, such as the Ca^2+^-activated K^+^ current [39] and M-type K^+^ current. Thus, our results imply that differences in the slow K^+^ current density might contribute to the response heterogeneity among ORNs.

### 3.2. Advantages of the proposed model

The model presented here serves as an efficient tool for simulating moth ORN responses. First, the model captures the typical response properties observed experimentally, particularly the phasic–tonic response pattern characterized by a rapid increase and a slow decay to a steady-state firing rate, as well as the effect of odorant concentration on the peak firing rate and first-spike latency. Second, our model can simulate cell-to-cell response variability among ORNs by individually setting only two parameters controlling the adaptive spike threshold. Third, our model provides the spike times, unlike linear–nonlinear models, which can capture only the firing rates [41,42,45]. Hence, our model could be useful for investigating the possibility of latency coding in olfactory information processing [46,47] and the role of spike-timing-dependent plasticity in olfaction [12,48,49]. Consequently, the proposed model can be applied to simulate a network of heterogeneous ORNs in order to investigate how ORN populations process olfactory information in the moth.

### 3.3. Limitations and future works

Experimental evidence suggests that adaptation occurs at the level of both the receptor potential and action potential generators [50,51]. This is effectively achieved in our model by including the chemical kinetics of activated receptors, which is dependent on the stimulation history, and by the adaptive threshold dependent on the spiking history. However, the proposed model does not consider detailed biochemical pathways downstream of odorant-receptor binding that also play a role in adaptive processes, since a comprehensive picture of the olfactory transduction does not emerge yet and since it is notoriously difficult to fit parameters of detailed biophysical models from limited experimental data. In such cases, even slight differences in initial parameter settings can lead to highly disparate results [52,53].

Sliding adjustment of odour response threshold and kinetics has several molecular actors, such as ion channels, second messengers and ORs. ORs make non-selective cation channels, which are permeable also for Ca^2+^. First, adaptation in *Drosophila* OR-expressing ORNs is mediated by the Ca^2+^ influx during odour responses [54] and Ca^2+^-dependent channels may also serve for odour adaptation as in vertebrate ORNs [55]. Second, G-protein signalling cascades can both increase or decrease the ORN sensitivity [56,57]. Finally, ORs also adjust their sensitivity according to previous odour detections [58,59]. Insect ORs are formed by an odour-specific OrX protein and an odorant co-receptor, Orco, which plays a central role in both downregulating and upregulating the ORN sensitivity. In moth pheromone-sensitive ORNs, Orco was proposed to function as a pacemaker channel, controlling the kinetics of the pheromone responses [60]. One or a combination of mechanisms of modulation of ORN sensitivity may contribute to expand the dynamic range of olfactory detection and thus allow the temporal structure of odour plumes to be encoded independent of their concentration [14].

In spite of its simplicity, our model effectively captures the adaptation process, since it can predict the response dynamics of ORNs recorded in experiments. However, the feedback mechanism of our model might be fundamentally different from that induced by the second messenger signalling pathways. For instance, the adaptation process due to the adaptive spike threshold model depends solely on previous spike history and is different from the adaptation process in real ORNs caused by Ca^2+^ influx and the following transduction cascade [7]. An investigation of more physiological feedback mechanisms could allow for further improvements of the model. One possibility may be to include explicit formulae describing the interaction of OR–Orco complexes and the adaptation of the rates of switching between the inactive and the active state, such as in the model by Gorur-Shandilya *et al.* [14].

## 4. Material and methods

### 4.1. Model of an olfactory receptor neuron

Here, we provide the details of the proposed neuron model.

#### 4.1.1. Receptor activation

Receptor activation by the pheromone (2.1)–(2.3) is described by the following reaction-rate equations:

4.1 $$\frac{\text{d}L}{\text{d}t}=\;k_{i}L_{\mathrm{air}}-nk_{1}L^{n}R+nk_{-1}R_{L}-k_{3}LN+k_{-3}N_{L},$$

4.2 $$\frac{\text{d}R}{\text{d}t}=\;-k_{1}L^{n}R+k_{-1}R_{L},$$

4.3 $$\frac{\text{d}R_{L}}{\text{d}t}=\;k_{1}L^{n}R-(k_{-1}+k_{2})R_{L}+k_{-2}R^{*},$$

4.4 $$\frac{\text{d}R^{*}}{\text{d}t}=\;k_{2}R_{L}-k_{-2}R^{*},$$

4.5 $$\frac{\text{d}N}{\text{d}t}=\;-k_{3}LN+(k_{-3}+k_{4})N_{L}$$

4.6 $$\mathrm{and}\;\;\frac{\text{d}N_{L}}{\text{d}t}=\;k_{3}LN-(k_{-3}+k_{4})N_{L},$$

where *k*_*i*_, *k*_1_, *k*_−1_, *k*_2_, *k*_−2_, *k*_3_, *k*_−3_ and *k*_4_ are the rate constants, *L*_air_ and L are the odorant concentrations in the air and in the sensillum lymph, respectively, *R*, *R*_*L*_ and R* are the concentrations of the receptors in the free, receptor–ligand complexed and activated states, respectively, *N* and *N_L_* are the deactivating enzyme concentrations in the free and complexed states, respectively. The total amounts of receptors R_tot_ and the deactivating enzyme N_tot_ do not change over time. Using

4.7 $$R_{L}=\;R_{\mathrm{tot}}-R-R^{*}$$

and

4.8 $$\;N_{L}=\;N_{\mathrm{tot}}-N,$$

the system of equations (4.1)–(4.6) can be reduced to

4.9 $$\frac{\text{d}L}{\text{d}t}=\;k_{i}L_{\mathrm{air}}-n(k_{1}L^{n}+k_{-1})R-nk_{-1}R^{*}-(k_{3}L+k_{-3})N+nk_{-1}R_{\mathrm{tot}}+k_{-3}N_{\mathrm{tot}},$$

4.10 $$\frac{\text{d}R}{\text{d}t}=\;-(k_{1}L^{n}+k_{-1})R-k_{-1}R^{*}+k_{-1}R_{\mathrm{tot}},$$

4.11 $$\frac{\text{d}R^{*}}{\text{d}t}=\;-k_{2}R-(k_{2}+k_{-2})R^{*}+k_{2}R_{\mathrm{tot}}$$

4.12 $$\mathrm{and}\;\frac{\text{d}N}{\text{d}t}=\;-(k_{3}L+k_{-3}+k_{4})N+(k_{-3}+k_{4})N_{\mathrm{tot}}.$$

The model parameters are listed in table 1.

#### 4.1.2. Spike generation

The membrane voltage *V*(*t*) of an ORN is described by the following equation:

4.13 $$C_{m}\frac{\text{d}V}{\text{d}t}=-g_{L}(V-E_{L})-\gamma R^{*}(t)(V-E_{R}),$$

where C_*m*_ is the cell capacitance, *g*_*L*_ is the leak conductance, *γ* is the unit receptor conductance, R*(*t*) is the concentration of activated receptor, and *E_L_* and *E_R_* are the reversal potentials of the leak and the receptor currents, respectively (parameter values shown in table 2).

The model neuron generates a spike when the voltage *V*(*t*) reaches the spike threshold *θ*(*t*), and, then, the voltage is instantaneously reset to a value *V*_reset_. We consider two descriptions for the threshold. In the first description, the threshold is constant, *θ*(*t*) = *θ*_0_. This description is equivalent to the standard LIF model [13,32]. In the second description, the spike threshold is modulated by previous spikes and is formally described as follows [33,35,36].

(1) When the neuron does not generate spikes, the threshold *θ*(*t*) decays exponentially to its asymptotic level *θ*_0_,
  4.14 $$\tau \frac{\text{d}\theta }{\text{d}t}=-(\theta -\theta_{0}).$$
  This implies that
  4.15 $$\theta (t)=\theta_{0}+[\theta (t_{\;f}^{+})-\theta_{0}]\;\text{exp}\left(-\frac{t-t_{\;f}}{\tau }\right),\;\text{for}\;t_{\;f}\leq t,$$
  where *t*_*f*_ is the time of the last spike and *t*^+^ represents the limit from above.
(2) If the voltage reaches the threshold at time *t*_*sp*_, *V*(*t*_*sp*_) ≥ *θ*(*t*_*sp*_), the threshold increases by a step Δ/*τ*, therefore
  4.16 $$\theta (t_{sp}^{+})=\theta (t_{sp}^{-})+\Delta /\tau ,$$

where Δ represents the strength of adaptation due to a single spike.

Equations (4.9)–(4.12), (4.13), (4.14) and (4.16) were solved numerically using the forward Euler integration method with a time step of 0.01 ms. The initial conditions were *R*(0) = *R*_tot_, *N*(0) = *N*_tot_, *V*(0) = *E_L_* and *θ*(0) = *θ*_0_, that is, all of the receptors and the degrading enzymes were in the free state, the voltage was at the resting value and the threshold was at the asymptotic level. The simulation code was written in R [61].

### 4.2. Experiments

*Insects*. Experiments were performed with laboratory-reared 4–5-day-old (sexually mature) adult male *Agrotis ipsilon* fed 20% sucrose solution *ad libitum* [62]. Pupae were sexed, and males and females were kept separately at 22°C under an inversed light–dark cycle (16–18 h light–dark photoperiod).

*Electrophysiology*. Insects were immobilized with the head protruding. One antenna was fixed with adhesive tape on a small support and a tungsten electrode (TW5-6; Science Products, Hofheim, Germany) was inserted at the base of a long pheromone-responding sensillum trichodeum located on an antennal branch. The reference electrode was inserted in the antennal stem. The electrical signal was amplified (×1000) and band-pass filtered (10 Hz to 5 kHz) with an ELC-03X (NPI electronic, Tamm, Germany), and sampled at 10 kHz by a 16-bit acquisition board (NI-9215; National Inst., Nanterre, France) under Labview (National Inst.). One sensillum was recorded per insect.

*Stimulation*. ORNs were stimulated with the major *A. ipsilon* sex pheromone, (Z)-7-dodecenyl acetate (Z7-12:Ac). Pheromone was diluted in decadic steps in hexane and applied to a filter paper introduced in a Pasteur pipette. The antenna was constantly superfused by a humidified and charcoal-filtered air stream (70 l · h^−1^). Air puffs (10 l · h^−1^) were delivered through a calibrated capillary (ref. 11762313; Fisher Scientific, France) positioned 1 mm from the antenna and containing the odorant-loaded filter paper (10 × 2 mm). An electrovalve (LHDA-1233215-H; Lee Company, France) was controlled by custom-made Labview programs reading sequences generated by Matlab scripts. The time resolution of the sequence was 1 ms. The characteristic response time of the valves, i.e. the time to switch from open to closed or closed to open, was less than 5 ms. The durations of the pheromone puffs and pauses were randomized. Time was divided into bins of a fixed duration (50 or 100 ms). In each bin, the probability of the valve being open was 0.5. Unique sequences of puffs and pauses were generated for each ORN. The dose of pheromone was constant throughout one recording session.

In total, recordings of 84 moth ORNs were obtained: 41 recordings with a 50 ms minimum puff/pause duration, 43 recordings with a 100 ms minimum puff/pause duration. Each combination of pheromone dose and minimum puff duration was tested on six or more ORNs. The first 100 s of each recording was discarded because the ORN activity was not stationary.

### 4.3. Parameter fitting

We first fitted the four parameters *n*, *γ*, *τ* and Δ to the average response time courses of ORNs under a pulse stimulation. For each odorant concentration, we extracted all recording segments where a neuron was stimulated with a puff longer than 0.5 s after a no-stimulation period longer than 0.1 s. Then we estimated the firing rate *f*(*t*) by convolving the spike train at the extracted segment with a Gaussian kernel function (standard deviation 0.03 s) [63,64]. The mean firing rate was calculated by aligning the individual firing rates with the stimulus onset and averaging across the cells stimulated by the same pheromone dose. The firing rate of the model neuron was obtained similarly by assuming a 0.5 s stimulation with the odorant concentration *L*_air_ equal to 0.1, 1, 10 and 100 pM that corresponds to the pheromone doses 1 pg, 10 pg, 100 pg, 1000 pg, respectively. The firing rate of the model was also calculated by convolving the spike train with a Gaussian kernel function (standard deviation 0.03 s).

The parameters *n*, *γ*, *τ*, Δ were tuned by minimizing the integrated square error

4.17 $$\epsilon_{\mathrm{ave}}^{2}=\sum_{L_{\mathrm{air}}}\int {\left(f_{d}(t|L_{\mathrm{air}})-f_{m}(t|L_{\mathrm{air}})\right)}^{2}\;\text{d}t,$$

where *f*_*d*_(*t*|*L*_air_) is the average firing rate for the experimental data, *f*_*m*_(*t*|*L*_air_) is the firing rate of the model and the summation was conducted across all concentrations of *L*_air_. The minimization was performed using the Nelder–Mead algorithm [65].

Subsequently, we fitted threshold parameters (Δ and *τ*) to the recording from each neuron. These parameters were tuned by minimizing the integrated square error in the 10 s training period

4.18 $$\epsilon_{\mathrm{ind}}^{2}=\int {\left(f_{d}(t)-f_{m}(t)\right)}^{2}\;\text{d}t,$$

where *f*_*d*_(*t*) is the firing rate of the recorded neuron and *f*_*m*_(*t*) is the firing rate of the model neuron. The model simulation was initiated 1 s before the start of the training period to reduce the influence of the initial conditions. Finally, the model performance was evaluated by the coefficient of determination in the subsequent 10 s prediction period. The coefficient of determination was defined as

4.19 $$R^{2}=1-\frac{\int {\left(f_{d}(t)-f_{m}(t)\right)}^{2}\;\text{d}t}{\int {\left(f_{d}(t)-\langle f_{d}\rangle \right)}^{2}\;\text{d}t},$$

where 〈*f*_*d*_〉 is the average firing rate of the experimental data.

## Data accessibility

Data and R code are available from GitHub at: https://github.com/MarieLevakova/Adaptive-integrate-and-fire-model.git.
